# Microscale Magnetic Field Modulation for Enhanced Capture and Distribution of Rare Circulating Tumor Cells

**DOI:** 10.1038/srep08745

**Published:** 2015-03-04

**Authors:** Peng Chen, Yu-Yen Huang, Kazunori Hoshino, John X.J. Zhang

**Affiliations:** 1 Department of Biomedical Engineering, University of Texas at Austin, Austin, TX 78712, USA; 2 Department of Biomedical Engineering, University of Connecticut, Storrs, CT 06269, USA; 3 Thayer School of Engineering, Dartmouth College, NH 03755, USA

## Abstract

Immunomagnetic assay combines the powers of the magnetic separation and biomarker recognition and has been an effective tool to perform rare Circulating Tumor Cells detection. Key factors associated with immunomagnetic assay include the capture rate, which indicates the sensitivity of the system, and distributions of target cells after capture, which impact the cell integrity and other biological properties that are critical to downstream analyses. Here we present a theoretical framework and technical approach to implement a microscale magnetic immunoassay through modulating local magnetic field towards enhanced capture and distribution of rare cancer cells. Through the design of a two-dimensional micromagnet array, we characterize the magnetic field generation and quantify the impact of the micromagnets on rare cell separation. Good agreement is achieved between the theory and experiments using a human colon cancer cell line (COLO205) as the capture targets.

Rare cell separation has been an important emerging process towards early diagnosis of diseases such as cancer^1^. In particular, Circulating Tumor Cells (CTCs), referring to the cells that have shed into the vasculature from a primary tumor site, and circulate in the bloodstream, have been demonstrated to be clinically significant due to its values in cancer diagnosis, prognosis and treatment monitoring^2^,^3^,^4^. The detection process usually involves the enrichment of the CTCs from interfering background hematocyte cells, before carrying on subsequent analyses^5^. To overcome the challenges of the natural rareness, a variety of approaches have been investigated towards efficient separation based on mechanisms such as adhesion^6^, filtration^7^, dielectrophoretic separation^8^, hydrodynamic manipulation^9^,^10^, and magnetic attraction^11^,^12^. Among these popular methods, the magnetic activated system in combination with immunoassay (also known as ‘immunomagnetic assay’) shows great potential, especially in its low detection limit, high sensitivity, specificity and throughput, which are all necessary for effective clinical applications^12^.

Immunomagnetic assay usually works by selectively labeling the target cells with magnetic tags through specific biomarkers, and using magnetic force generated by permanent magnets to drive the cells for separation. It has been widely used for cell detecting, sorting and manipulating^13^,^14^,^15^,^16^, as summarized in previous review^17^. However, in traditional immunomagnetic assays, the efficacy of the magnetic field generated by permanent magnets (usually in the scales of centimeter or millimeter) is limited by the low value of magnetic field gradient and the low density of traps. Consequently, the target cells and magnetic tags tend to be captured and aggregated in a confined area. The aggregation may directly impact the structural integrity or quench the fluorescent signals from the target cells, all of which may interfere with cell imaging, identifying and weaken the strength of this approach. We propose a potential solution to the aggregation issue by modulating the in-channel magnetic field through implementing microscale magnetic structures – ‘micromagnets’, which are designed to generate localized strong magnetic field gradient upon magnetization and create multiple distributed capture sites.

Modulating magnetic field is critical in a variety of applications, such as cell proliferation regulating^18^, magnetic particle trapping and manipulating^19^,^20^,^21^, and chemical kinetic modulation^22^,^23^. It usually associates with precise confinement of the magnitude and distribution of the magnetic field and gradient. As for separation purposes, several early studies have been reported on the integration of micromagnets with microfluidic systems. For example, nickel micro-strips have been fabricated to separate leukocytes from whole human blood as magnetic tracks^24^. Arrays of nickel posts are used in a microfiltration device to separate magnetic beads from non-magnetic beads^25^. Shrink-induced magnetic traps are used to extract DNA samples for qPCR studies^26^. Thermomagnetically patterned micromagnets are used to separate magnetic and non-magnetic micro-particles from a mixed solution^27^,^28^.

However, for rare cancer cell studies, the aforementioned micromagnet structures might not serve the purpose. Since the cancer cells are rather fragile^29^, the relatively large thickness (>5 μm) of the previous structures might cause physical damages to the cells due to collisions. Therefore, we pursue an ultra-thin structure with sub-micrometer thickness to minimize possible damages to the cells. Additionally, in the demonstrated applications using aforementioned micromagnets to sort targets with large sub-populations, such as white/red blood cells^24^, magnetic/non-magnetic microbeads^27^,^28^, separation efficiency is the major key parameter that matters. However, when it comes to rare cell studies, each captured target cell needs to be individually addressable, structurally distinguishable, fluorescently visible, and potentially retrievable to facilitate downstream analyses. It posts extra requirements on avoiding cell aggregation. Therefore, we adopt an array design, anticipating the array captures cells discretely and provide a promising tool to generate better distribution of the captured CTCs.

In the proposed device, we take a multi-dimensional approach – using permanent magnets for a long-range attraction, and using thin-film micromagnets for short-range retaining. Since magnetic field gradient increases as the size of the magnetic source downscaled, the interactions between target cells and magnetic field can be significantly enhanced on the channel substrate due to the ferromagnetic micromagnets. The patterned thin-film micromagnet approach is also appealing in that the magnetic field enhancement can be realized at ultimately single cell resolution, and can be well controlled by adjusting the geometries, materials, and distributions of the micromagnets during the design and fabrication stages. More importantly, considering the small size of the micromagnets, they can be easily implemented into most of the current immunomagnetic assays seamlessly without affecting other functional components or sacrificing the system throughput.

In this paper, we demonstrate the enhanced capture and distribution for CTCs detection by modulating the surface magnetic field with low-profile microscale magnetic structures. We first present the theoretical framework and analytical models to describe the physics of the thin-film micromagnet array implementation. The model is described with more details below in the Methods section. Briefly, a two-dimensional micromagnet array model is taken to characterize the magnetic field generated by the micromagnet elements, which also helps define key parameters such as the effective range. In addition, we build a two-dimensional model simulating the immunomagnetic assay, and investigate the impact of the micromagnets on rare CTCs detection with primary focus on altering the cell distribution. Then, to validate our models, we perform screening experiments using thin film micromagnets for the enrichment of COLO205 cells (a type of human colorectal cancer cell line) from whole blood samples. Previous studies have revealed the similarities between cultured cancer cells and CTCs from patients in terms of morphology^30^ and biomarker^5^. Therefore, cultured cancer cells have been widely used to demonstrate CTC detection system, including PC3, SKBR-3, T-24, MCF-7, and COLO205^5^,^6^,^31^,^32^,^33^. Here we choose a colorectal cancer cell line (COLO205) as our separation target.

## Results

### Thin-film micromagnet design, magnetization and integration with immunomagnetic assay

**Figure 1:**
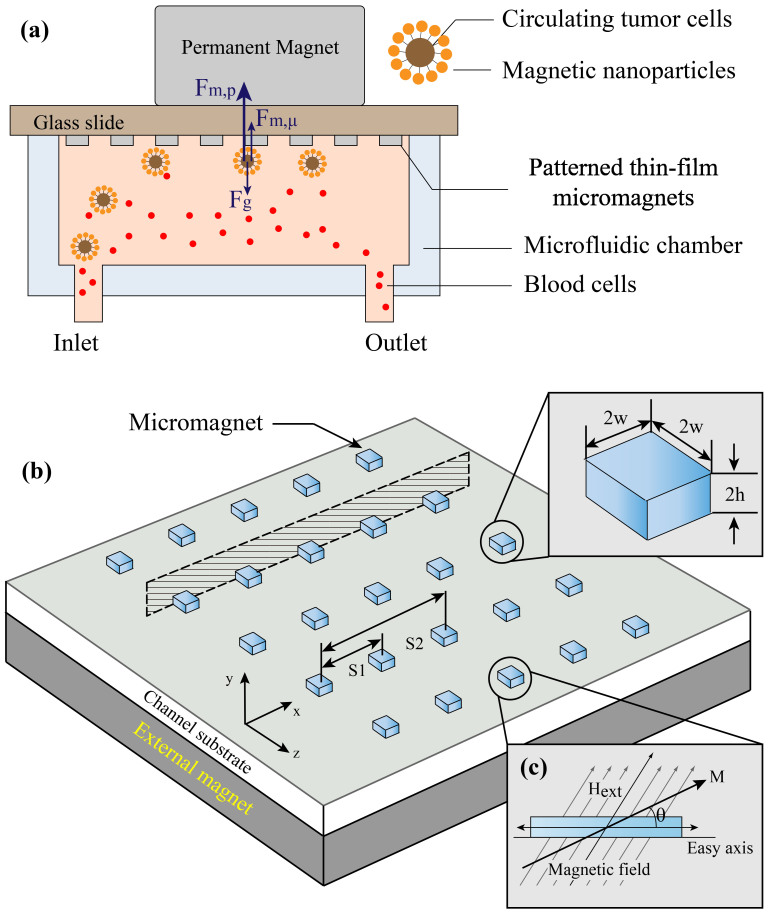
Schematic of the patterned thin-film micromagnet integration, design and magnetization process. (a) Schematic of the microchip based immunomagnetic assay integrated with micromagnets, and the major forces applied on the cells when they are on the substrate. (b) Concept of the micromagnet design - after being magnetized by the external magnetic field, the ferromagnetic micromagnets generate strong localized field to increase the magnetic force applied on the target cells. (c) Magnetization process of the soft-magnetic micromagnets using the theory of magnetic anisotropy and demagnetizing field.

The concept of the thin-film micromagnet integrated with a microfluidic based immunomagnetic assay is illustrated in Figure 1(a), where ferromagnetic thin films are patterned on the substrate of a microchannel. Target CTCs are labeled with magnetic nanoparticles based on cancer specific antibodies. When the blood sample flows through the microchannel, CTCs are attracted by the permanent magnets, which are placed outside the channel, and trapped by the thin film micromagnets on the channel substrate while normal blood cells are unaffected and flow out of the channel. It is noteworthy that during the experiments, the microchannel is placed in an inverted orientation to take advantage of the gravity in separating the CTCs from blood cells^34^.

Specifically, the geometries and layouts of the micromagnet array can be defined using microfabrication techniques such as photolithography. The entire device is placed on top of a permanent magnets array. After being magnetized by the external magnetic field, the ferromagnetic element generates strong localized field to increase the magnetic force applied on the target cells, as shown in Figure 1(b).

The magnetization process of soft magnetic materials has been well established using the concept of magnetic anisotropy and demagnetizing field, as shown in Figure 1(c)^35^. Briefly, upon application of an external magnetic field 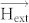 with an angle to the net magnetization 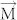 of the sample, 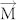 is rotated by the torque exerted by 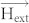 with an angle *θ* from the equilibrium direction, which is usually the long axis. In a permanent magnet analysis, for magnetic structure with large aspect ratio (our case 20 μm:200 nm = 100:1), shape induced anisotropy plays a dominate role in the magnetization process. Therefore, the assumption *θ = 0* is made to simplify the calculation, only magnetization parallel to the long axis is considered. We incorporate the magnetic anisotropy theory to describe the magnetization process induced by the in-plane component of the external magnetic field, which is parallel to the micromagnets.

### Magnetic field and effective range characterization of the thin-film micromagnets

We first calculate the magnetic field generated by a single micromagnet element. In our system, the micromagnets are made with nickel (Ni). The dimension of each micromagnet is 20 μm (width) × 200 nm (thickness). The saturation magnetization of nickel is found to be 55.1 *emu/g*^36^ (can be converted to volume magnetization of M_es_ = 4.9 × 10^5^ *A·m*^−1^). We plot the distribution of both X and Y components of the magnetic field along horizontal lines (−40 μm < x < 40 μm), at three different heights (h = 5 μm, 10 μm and 15 μm) on top of the micromagnet.

**Figure 2:**
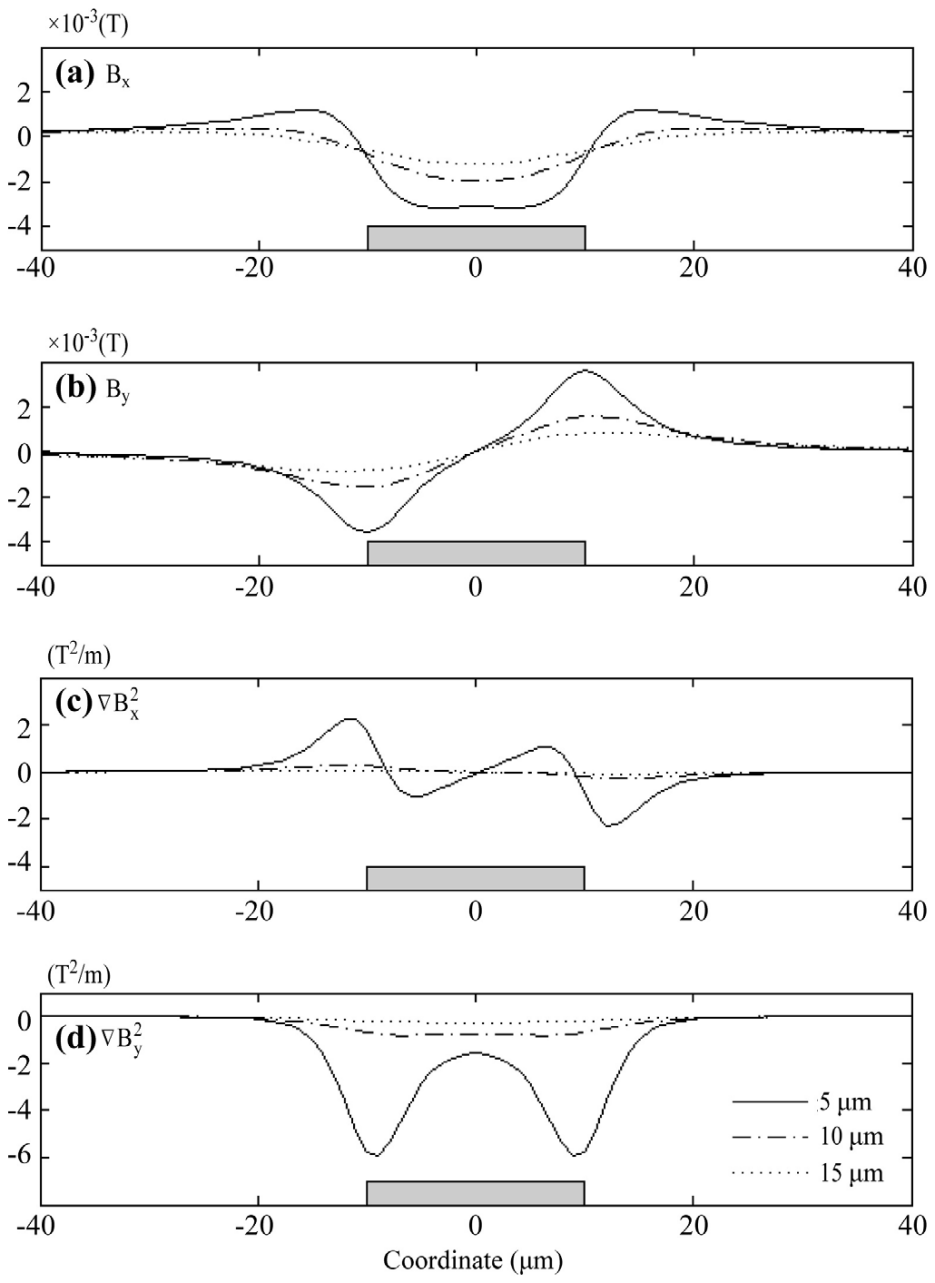
Magnetic field and gradient calculated for a single micromagnet element (a) B_x_, (b) B_y_, and the magnetic field gradient (c) 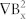 and (d) 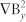. The gray box indicates the positions of the micromagnet. The curves are plotted along horizontal lines (from −40 μm to 40 μm) with different heights (h = 5 μm, 10 μm and 15 μm) on top of the micromagnet.

The results can be seen in Figure 2 ((a) *B_x_* (b) *B_y_*), in which the micromagnet is shown as a gray box to indicate the position (size is not to scale). The magnetic field curves are shape-coded according to the heights of the plotting. The magnetic field generated by the micromagnet decays fast in both vertical and horizontal directions. Since the value of magnetic force is largely dependent on the properties of the targets, we study the gradient 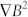 as an indirect but objective measurement of the magnetic force. We plot the magnetic field gradient (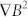) at the same heights. The results are presented in Figure 2(c) (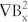) and Figure 2(d) (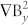). The negative values of the gradient in Y direction indicate that the micromagnet generates attractive force towards the micromagnet that helps retaining the cells.

To calibrate the strength of the micromagnets, we define the effective range of a micromagnet element as the distance where magnetic force acting on the cells is equivalent to the gravitational force. As shown in Figure 1(a), when the cells are on the bottom, they are driven by the magnetic forces from the permanent magnets (*F_m,p_*) and micromagnets (*F_m,μ_*), and the gravitational force (*F_g_*). To simplify the calculation, friction force between the cells and the channel surface is neglected^37^. The magnetic force generated by the permanent magnets overweighs the other two, and dominates the motion of the cancer cells^34^. Therefore, to investigate the effect of the micromagnets solely, we make the comparison between the gravitational force and the magnetic force provided by the micromagnets. According to our previous calculations, the gravitational force on a single cancer cell is *F_g_* = 1.3 × 10^−12^[*N*]^34^, and an equivalent magnetic force determines the threshold magnetic gradient to be 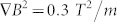. Therefore, the vertical effective range (in Y direction) is determined to be 15 μm. As for the lateral range (in X direction), we make the decision by examining the gradient at 10 μm on top of the micromagnets, which corresponds to the center of the CTCs when trapped on the surface (typical diameter of CTCs is ~20 μm)^7^. The lateral range is found to be ±15 μm. The fact that the effective capture range of the micromagnet is comparable to the size of a single cancer cell indicates that only a few cells can interact fully with each micromagnet through its magnetic field. It helps distribute cell populations across the substrate covered by the network of micromagnet elements.

CTC clusters were discovered in the blood of cancer patients, which might be clinically important, but the mechanism behind it has not been fully understood^38^. Compared to single cell, a cluster is easier to capture (refer to the derivation for the cell cluster motion in the Methods section), and may cause errors in cell identification and counting. To break the cell clusters, the cell suspension is mixed and incubated with trypsin, which works by cleaving bonding proteins, until over 90% of the cells are individually dispersed. Then the cell suspension is spiked into the blood sample.

**Figure 3:**
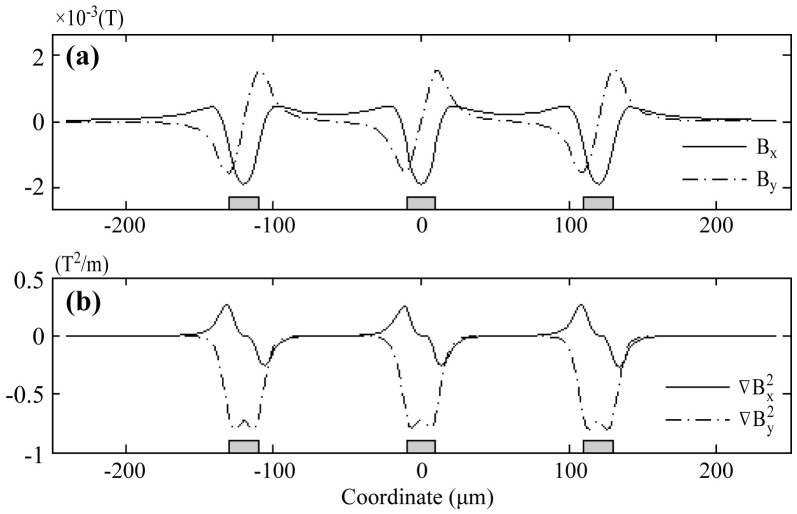
Magnetic field (a) and magnetic field gradient (b) of a linear array of three micromagnets. Figures are plotted 10 μm on top of the micromagnet array.

In addition, we perform calculations for micromagnet array (an array of 3 elements linearly aligned is used as an example), with the magnetization along the horizontal direction. The dimensions of each micromagnet are consistent with the single element study (20 μm × 200 nm), and the edge-to-edge distance between adjacent micromagnets is 100 μm. The magnetic field (X/Y components) along a horizontal line 10 μm on top of the micromagnet array is shown in Figure 3(a). The X/Y components of the gradient are shown in Figure 3(b). The magnetic field and gradient generated by micromagnet array exhibits the same characteristics as single element, with the profiles being spatial repetition of single micromagnet.

### Impact of the micromagnet array on the rare CTC separation

**Figure 4:**
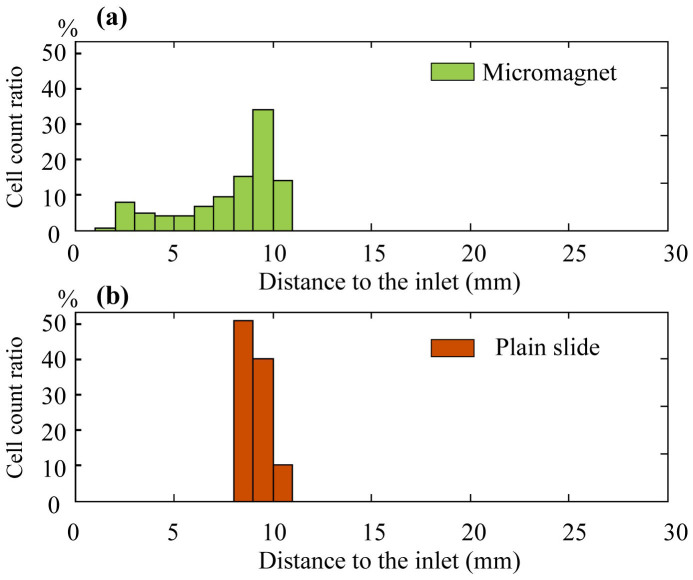
Simulation results of the imapcts of micromagnets on rare cell detection. Distribution histogram of the captured CTCs of (a) micromagnet slide and (b) plain slide.

To investigate the impacts of the thin-film micromagnet array on rare CTCs separation, we incorporate the micromagnets into a two-dimensional theoretical model simulating a microchip based immunomagnetic assay and trace the movements of the target cells. Key design parameters of the microchip, and detailed introduction are included in the Methods section. We divide the motion of the target cells into two stages - (i) in free space and (ii) on solid surface after hitting the channel substrate to obtain the final locations of the captured cancer/target cells. More details about the cell tracking algorithm can be found in the Methods section. In the first stage, the cells are attracted by the permanent magnets and gradually move towards the substrate as they flow through the channel. In the second stage, cells continue moving under the combined influence of the permanent magnets and micromagnets. The calculated histograms of the final positions of the captured cells are shown in Figure 4(a), (b) for micromagnet and plain slide (standard glass slide) separately. On plain slide, most of the cells are captured and aggregated in a confined area, where the front edge of the permanent magnet lies (refer to the paragraph about the immunomagnetic assay model in the Methods section for the position of the permanent magnet). In contrast, micromagnet slide exhibits a broader cell distribution spectrum from the inlet to the front edge of the permanent magnets as expected. We calculate the average distance of all the captured cells to the inlet, and make the comparison between the plain slides and micromagnet slides. Based on the simulation data, the average distance on plain slide is 9.2 ± 0.7 mm (n = 150), and 7.9 ± 2.5 mm (n = 150) on micromagnet slides. Compared to the plain slides, the micromagnets change the average distance. The simulation results indicate that the micromagnet array re-arranges the magnetic field inside the microchannel and improves the distribution patterns of the captured CTCs.

### Experimental results of separating CTCs from blood samples

**Figure 5:**
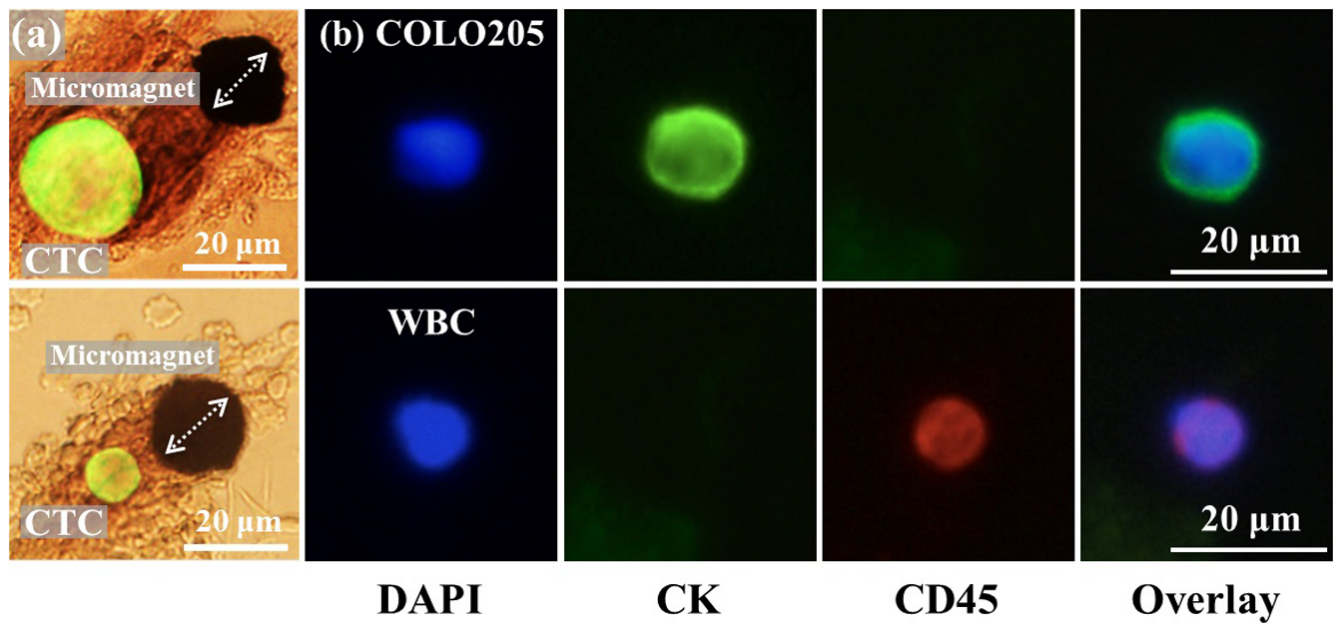
Immunomagnetic CTC screening experimental results. (a) Observation of COLO205 cell being captured by the micromagnet with bright field microscope confirms the attractive interactions between CTCs and the micromagnet. The aggregation patterns of the magnetic nanoparticles can help estimate the direction of the magnetization of the micromagnets, as indicated by the arrows. (b) Fluorescent signal of the CTCs and WBCs of three dyes (DAPI, CK, CD45). CTCs are DAPI+, CK+ and CD45−, and WBCs are DAPI+, CK− and CD45+.

To verify the simulation results, we carry out screening experiments to separate cultured COLO205 cells (human colorectal cancer cell line) from blood samples. After the screening process, the experimental slides are stained with three fluorescent dyes (DAPI, anti-cytokeratin and anti-CD45) targeting different cellular components for identification. Details of the experimental protocol are reported in the Methods section. We could directly observe the target cells being captured by the micromagnets under bright field microscope, as shown in Figure 5(a), which confirms the magnetic attractive interactions between the micromagnets and the CTCs. In Figure 5(a), we could observe some roughness at the edges of the micromagnets. These small structures could further increase the magnetic field gradient nearby. However, considering their small size (~2 μm) compared to the size of a cancer cell or a micromagnet, the effect of the roughness on the cell separation can be neglected. In addition, we could observe the aggregation of the magnetic nanoparticles around the micromagnets, as is shown in the “brown stains” in Figure 5(a). The directional aggregation pattern provides a simple clue to estimate the direction of the magnetization of the micromagnets, as shown by the arrows in Figure 5(a). Besides, the aggregated nanoparticles, as magnetic materials themselves, can potentially increase the effective range of the micromagnets. Figure 5(b) shows the fluorescent images of a captured COLO205 cells (DAPI+, CK+ and CD45−) and one white blood cell (DAPI+, CK− and CD45+) for comparison.

**Figure 6:**
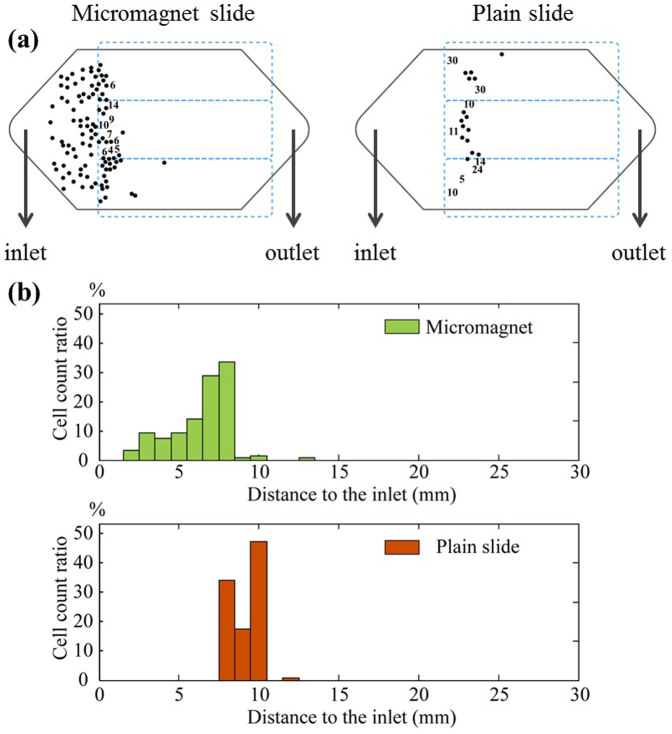
Experimental results of spiked COLO205 cells. (a) Top-view locations of the captured COLO205 cells on plain slide and micromagnet slide, respectively. The black hexagon boxes represent the microchannels, and the blue boxes indicate the positions of the permanent magnets. (b) Distribution histogram of the captured CTCs from micromagnet slide and plain slide, respectively.

**Figure 7:**
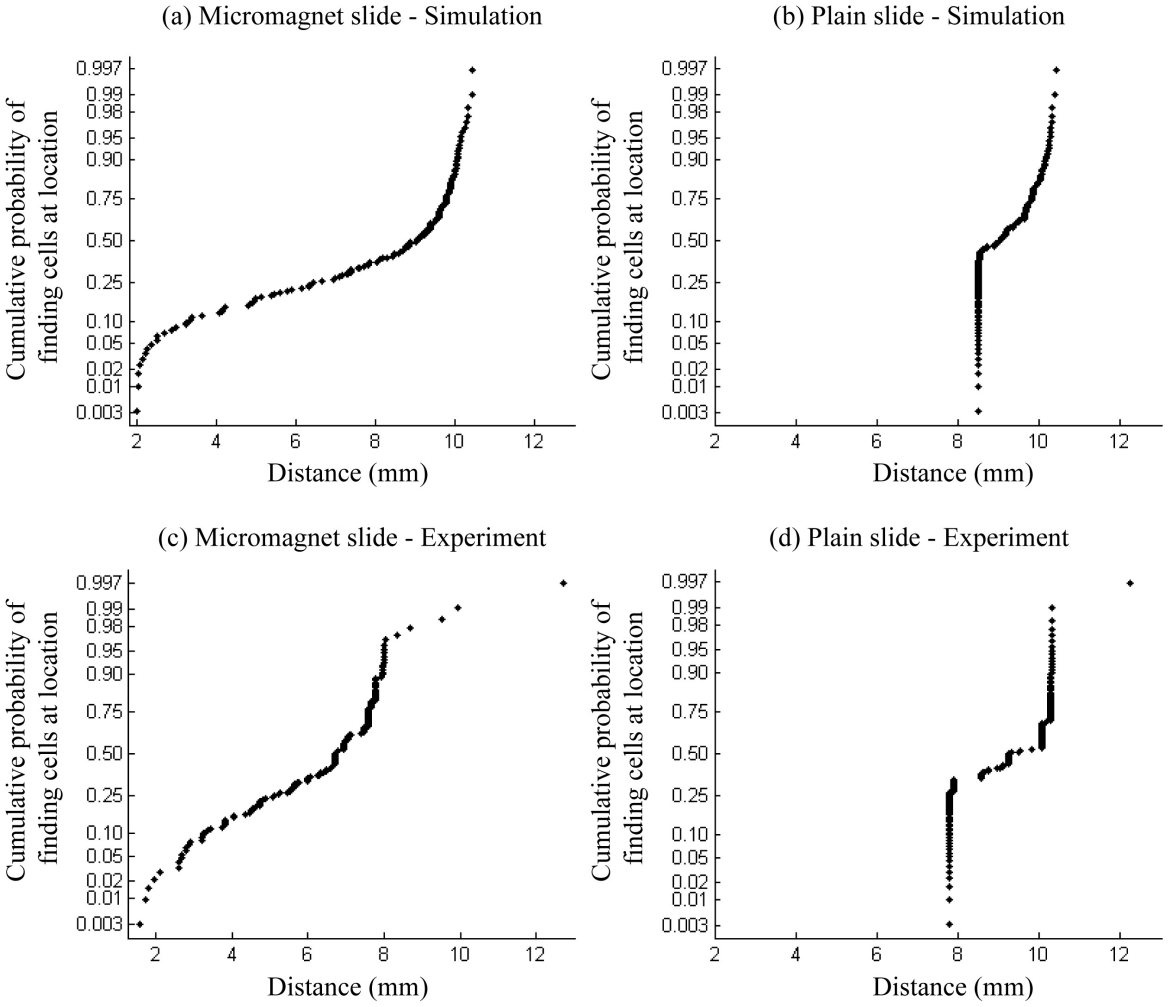
Normal probability plots of the cumulative probability of finding cells at certain locations from micromagnet and plain slides, respectively. (a), (c) are the plots with micromagnet slides, both showing a left skewed distribution with larger variance. (b), (d) are the plots with plain slides, showing a narrow distribution in a confined area.

We run experiments using micromagnet slides and plain slides in parallel for comparisons. Figure 6(a) shows example location maps of the captured CTCs on the micromagnet and plain slide respectively. The black hexagon represents the top view of the microchannel, and the blue dash boxes represent the permanent magnets. Please note that the numbers in Figure 6(a) indicates the number of cells found within one field-of-view of the microscope at the corresponding location. They are too close to be individually marked using the dots on the cell distribution map. We measure the positions of the cells based on the distance to the channel inlet and generate the distribution histogram in Figure 6(b). The average distance is 9.2 ± 1.1 mm (n = 129) on the plain slides, and 6.3 ± 1.8 mm (n = 151) on the micromagnet slides. The micromagnets significantly change the average distance (p = 2e-20 < 0.05) based on a two-sample T test and increase the variance (p = 4.2e-6 < 0.05) based on a Levene's test. To better show the strong correlations between the simulation and the experimental results, we make the normal probability plot based on the cell locations, as summarized in Figure 7. Both plots with micromagnet slides show left skewed distribution with large variance. While both plain slides show narrow distribution in a very confined area. The experimental data well verify the more spread out distribution of the cells after the implementation of the micromangets as predicted by the simulations.

To quantify the comparison of the cell distribution, we define a parameter “distribution uniformity” by calculating the number of unit area (1 mm × 1 mm) within the microchannel, which has at least one captured cell to reflect the channel space occupancy. Based on the surface area of the microchamber, about 350 unit areas are used for the calculation. On plain slides, the uniformity is found to be 9 ± 4% (n = 4), while after the implementation, the micromagnets change this value to 23 ± 4% (n = 4). The pattern-integrated chip yields a significantly improved distribution uniformity in comparison with plain chip by 14% on average (p = 0.0044 < 0.05). Meanwhile, we study the capture rates before and after the implementation of the micromagnets. The capture rate for plain slides is 79 ± 18% (n = 15), and for patterned slides is 98 ± 9% (n = 15). The integration of the patterns significantly increases the capture rate by 19% on average (p = 0.0012 < 0.05).

## Discussion

The thin-film micromagnet integration improves the immunomagnetic cell separation from two perspectives. Firstly, as individual ferromagnetic piece, each micromagnet generates extra short-ranged magnetic force to retain the CTCs on the surface. It helps reduce the chances of losing cells and yield higher capture rates. Secondly, as an array, it creates discontinuous capture sites to alleviate the harmful aggregation issues of the immunomagnetic assay. Consequently, it lowers the chance of fluorescent quenching and facilitates the cell imaging, identifying and counting.

To adapt the micromagnet towards different applications, the geometry needs to be carefully tailored. Key parameters include lateral dimension (2 × w), thickness (2 × h) of single micromagnet element and spatial periodicity of the micromagnet array (s_1_, *s*_2_...), as illustrated in Figure 1. The lateral dimension determines the effective range of individual micromagnet. Larger the size, broader the area get affected. Using advanced fabrication techniques, such as E-beam lithography, the lateral dimension of the micromagnet can be even reduced down to nano-scale. However, given the size of the target CTCs (~20 μm), nano-scale magnets are not strong enough to hold the cells. Meanwhile, thickness affects the system performance both hydrodynamically and magnetically. On one hand, in-channel structures can be used to disturb the hydrodynamic flow in the channel, creating additional vertical flows as a micro-scale mixer, which increase the interactions between the cells and substrate for better capture^38^,^39^. However, thick micromagnets increase the shear stress that might cause damage to the cells. On the other hand, thickness also determines the magnitude of the magnetic force and the effective range of the micromagnets. As an array, the spatial periodicity can be engineered to adjust the distribution of the captured CTCs. Appropriate spatial periodicity is mainly determined by the effective range of single micromagnet element. Beyond this value, cells are stagnated at the entrance of the micromagnet array. Whereas below this value, the chances of cells hitting upon a micromagnet in their trajectories become so low that the effect of the micromagnets can almost be neglected. These factors need to be well balanced in searching for an optimum micromagnet design.

In this paper, we present the design, modeling and analyses of a microscale magnetic immunoassay, using patterned thin-film micromagnet array to modulate local magnetic field towards CTC capture with enhanced sensitivity and distribution. We calculate the magnetic field generated by the thin film micromagnets, and determine the vertical effective range to be 15 μm, and lateral range ±15 μm. To investigate the impact of the micromagnet array on altering the distribution of the captured cells, we incorporate the micromagnets into a 2D model simulating the immunomagnetic assay and observe an extended distribution spectrum. We also carry out experiments with COLO205 cells to validate the simulations. In the comparison between micromagnet and plain slides, we observe an average 19% increase in capture rate, and 14% increase in distribution uniformity. The thin-film micromagnets design enables effective immunomagnetic assay for rare cell studies.

## Methods

### Calculation of the magnetic field generated by the thin-film micromagnets

**Figure 8:**
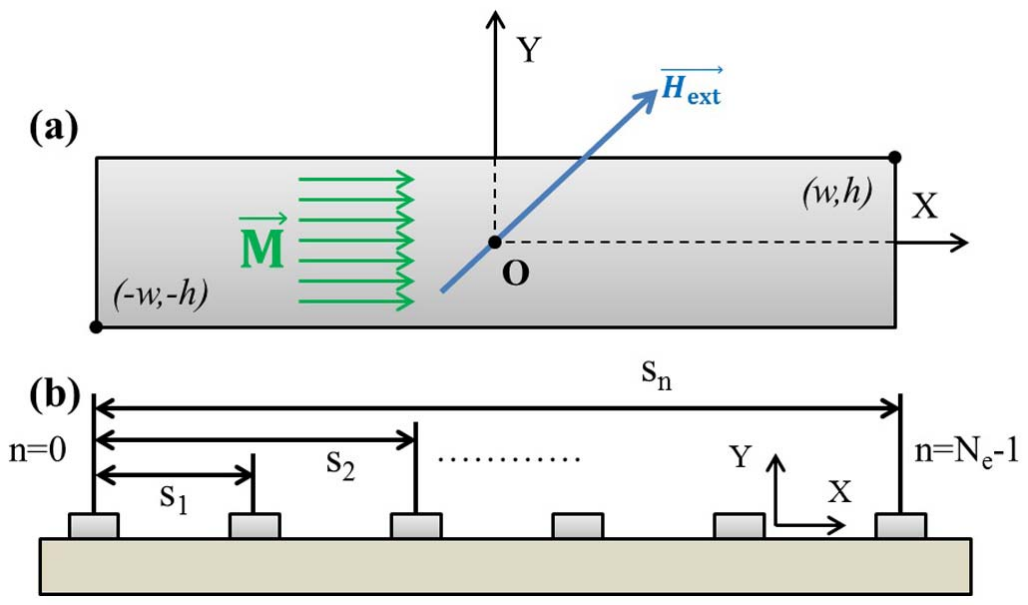
Analytical models to calculate the magnetic field generated by the thin-film micromagnets. (a) a single micromagnet element magnetized along the x axis. (b) an array of micromagnet elements aligned linearly on the substrate, with certain periodicity.

To quantify the strength of the our thin film micromagnets, we adopt an analytical model to describe the magnetic field generated by soft magnetic structure with rectangular shape^40^. In a simplified two-dimensional model (simulating the cross-section of the micromagnets, as shown in Figure 8(a)), a rectangular element (width 2 × w, height 2 × h) centering with respect to the origin in the x–y plane is magnetized by an external magnetic field 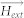. Assuming 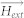 is strong enough to magnetize the micromagnet to saturation (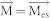, 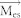 is the saturation magnetization of the material) along the X direction, the magnetic field generated by this soft magnetic element can be calculated as:
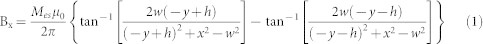

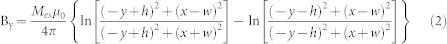
Here μ_0_ is the magnetic permeability of vacuum. Please note that we modify the magnetization direction and geometry notations from the original equations in order to be consistent.

Equation (1), (2) can be used to calculate the magnetic field generated by a single micromagnet. For micromagnet array, the total magnetic field can be computed based on super-position theory, i.e. the total magnetic field is the linear summation of magnetic field generated by each micromagnet within the array. Consider an array of *N_e_* elements (indexed with n = 0, 1, 2, 3… N_e_ − 1) with the first element centered with respect to the origin, and all the other micromagnets linearly positioned along the x-axis with distance s_n_ to the first element, as illustrated in Figure 8(b). The n^th^ element is centered at x = s_n_ on the x-axis, whose magnetic field can be calculated by shifting the coordinate system used in the 0^th^ component as follows:
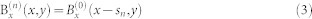

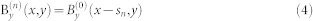
Please note that Figure 8(b) is essentially the cross-section shown in Figure 1(b). Eventually, the total field of the element array can be obtained by adding up the field components from all the micromagnet elements,
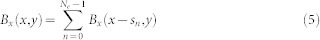

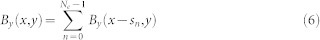


### Magnetic separation and cell tracking algorithm

In order to investigate the influence of the micromagnets on the separation of rare cells, we build a theoretical model to trace the target cells. We divide the motion of the target cells into two stages - (i) in free space and (ii) on solid surface after hitting the channel substrate. In the first stage, permanent magnets provide the primary long-range attraction. Motions of a single cell is determined by the magnetic force (*F_m,cell_*) and the drag force (*F_d,cell_*), each can be calculated as^13^,^34^:
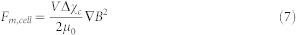

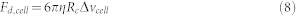
Here *η* is the medium viscosity, *R_c_* is the radius of the cells, Δ*v_cell_* is the relative cell velocity to the medium, *μ*_0_ is the magnetic permeability of vacuum, *B* is the magnetic field intensity, and 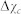 is the effective magnetic susceptibility of the cells, which is given by 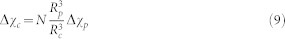
Here *R_p_* is the radius of the magnetic nanoparticles used to label the cells, 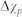 is the magnetic susceptibility of the nanoparticle, and N is the number of nanoparticles per cell. Assuming the cells are in quasi-static motion, which equates the drag force to the magnetic force (*F_d,cell_* = *F_m,cell_*), the relative velocity of a single cell can be represented as: 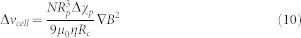
The final velocity of a single cell is the vector addition of the flow velocity (*v_flow_*) and the relative velocity: 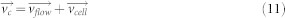
In the second stage, cells are already on the substrate of the microchannel and continue moving. To determine the final capture locations, we set up the criteria that if the value of the velocity perpendicular to the substrate 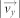 became larger than the component parallel to the substrate 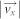 with a factor *λ* (Equation (12)), the cells are fully stopped because the forces on the cells tend to stop them more than pushing forward.
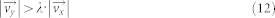
Otherwise, the cells keep moving on the substrate until the condition in Equation (12) is met^24^. Therefore, the short-range retaining force provided by the micromagnets essentially increases the value of 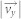, creating additional sites that meet these criteria for a firm capture.

**Motion of the cell cluster**. Considering a cell cluster contains *n* single cells, the magnetic force applied *F_m,cluster_* on the cluster is: 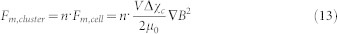
The drag force is dependent on the effective hydrodynamic size of the cluster, and can be computed as: 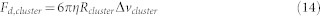
Under the same quasi-static motion assumption (*F_d,cluster_* = *F_m,cluster_*), the relative velocity of the cluster is:
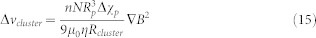
Based on the relation between a cluster and a single cell in their effective sizes *R_cluster_* < *n · R_c_*, the relative velocity of the cluster is always larger than the value of a single cell:
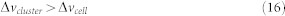
Equation (16) indicates that the cell cluster is more responsive to the magnetic attraction and is expected to be captured more easily.

Parameters used in the calculations are: 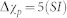, *R_p_* = 50 *nm*, *R_c_* = 7.5 *μm*, *η* = 10^−3^ *kg*·*m*^−1^·*s*^−1^, *μ*_0_ = 4*π* × 10^−7^ *T·m·A*^−1^, flow rate *Q* = 2.5 *ml*/*hr*, the number of particles per cell *N* = 2500 ~ 3500. For the stopping factor, we first find λ = 1.2 to match the theoretical and experimental results of the plain slides, and then the same value is used for the micromagnet patterned slides.

### Immunomagnetic assay model

**Figure 9:**
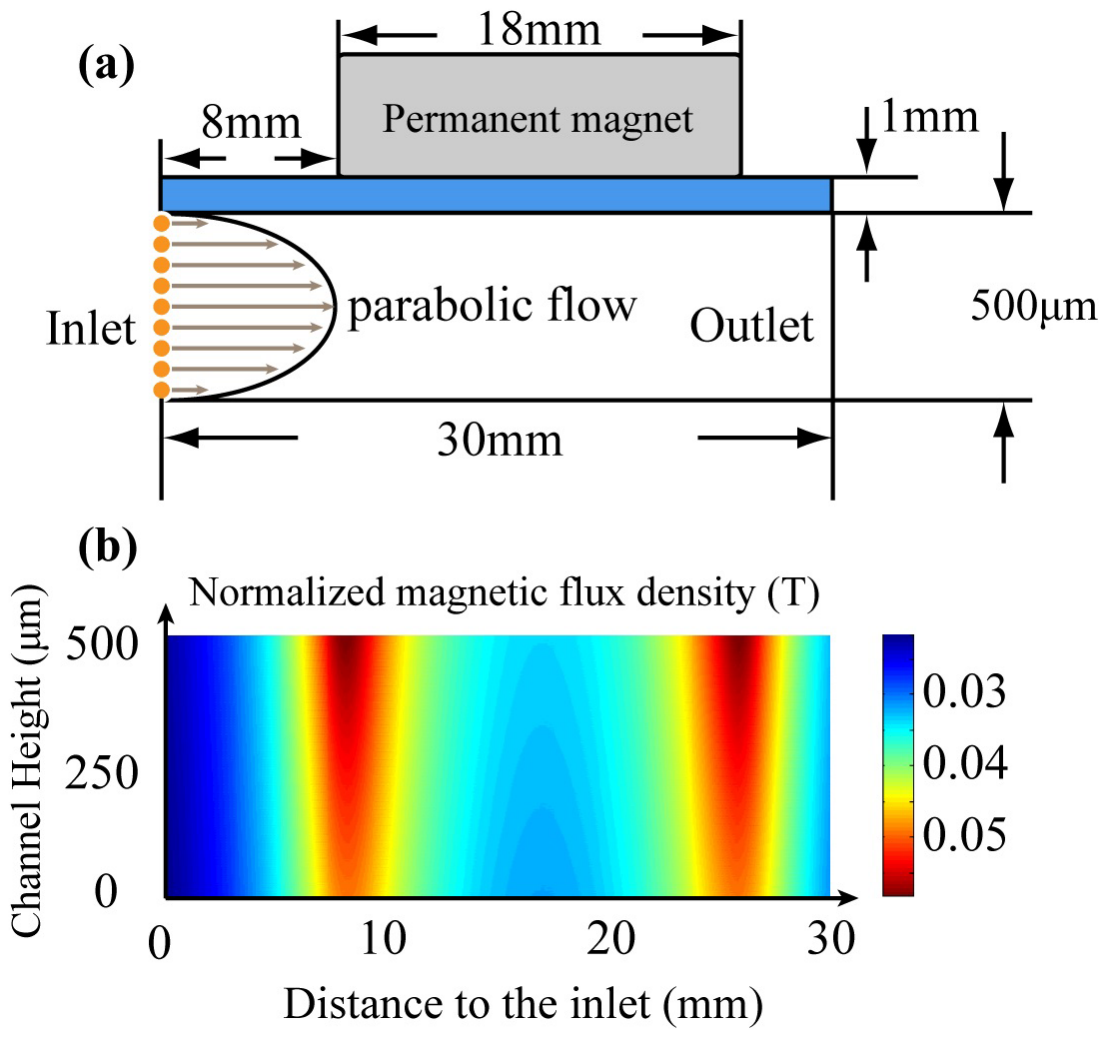
Analytical model of the microchip based immunomagnetic assay. (a) 2D model represents the microchip for rare cells detection. Liquid flow inside the channel follows standard parabolic profile. Cells are released from one side of the channel with uniform initial positions. (b) Magnetic field inside the microchannel generated by the permanent magnets. The area displayed is corresponding to the space inside the microchannel (30 mm × 500 μm).

The two-dimensional model representing cross-section of the microchip based immunomagnetic assay is shown in Figure 9(a). The dimensions of the model are identical to those of the real device (channel height = 500 μm, length = 30 mm). Cells are released from left side of the channel, with initial positions uniformly aligned from the bottom to the top of the microchannel. The cell count is set to be 150 to match the value in the spiked experiments. The flow rate is selected to match the experiment condition 2.5 ml/hr. Flow field inside the microchannel follows a standard parabolic flow profile. In Figure 9(a), the gray box represents the permanent magnets, with a length of 18 mm. The normalized magnetic flux density generated by the permanent magnets inside the microchannel is calculated using FEM simulation software, and is shown in Figure 9(b). The magnetic field is getting stronger when it is close to the permanent magnets, especially the edges of the permanent magnets.

### Micromagnet fabrication

The micromagnets can be easily fabricated using standard microfabrication techniques. Firstly, the photoresist is spin-coated onto the standard glass slide. Patterns of the micromagnets are then defined on photoresist by selective exposure to UV light through a photo-mask. Next, chromium layer is deposited as the adhesion layer, after which the nickel layer is deposited to form the micromagnet structure. In the final step, lift-off technique is used to remove the photoresist and leaving behind the micromagnets array. Detailed fabrication process of the micromagnets is introduced in our previous report^41^. Based on the total surface area of the microchamber and the periodicity of the micromagnet array, the total number of micromagnet element on one chip is estimated to be ~8750, about 25 elements per mm^2^.

### Cell screening experiment protocol

The COLO205 cell suspension is first mixed and incubated with trypsin (0.05% Trypsin-EDTA (1X), Phenol Red, Life Technology) for 5 minutes to break the cell clusters and ensure the cells flow through the microchannel individually. Observe the cells under the microscope until over 90% of the cells are individually dispersed, otherwise increase the incubation time a few more minutes, and check for dissociation every 30 seconds. Then same amount of cell culture medium (RPMI 1640 with 5% fetal bovine serum) is added to the suspension to neutralize the trypsin. A cell suspension (10 ~ 20 μL) containing approximately ~150 cells is spiked into 2.5 mL aliquot of blood sample acquired from healthy donors. Then, magnetic nanoparticles (*Ferrofluid^TM^, Janssen Diagnostic, LLC*), which are functionalized with cancer specific antibodies *anti-EpCAM*, are added to the blood samples to label the COLO205 cells. The typical diameter of this particle is around 100 nm. PBS is used to fill the microchannel before introducing the blood sample to eject air bubbles at the flow rate of 5 ml/hr. The blood sample is then driven through the microchannel at a flow rate of 2.5 ml/hr with a syringe pump. After the screening, PBS is introduced to wash the remaining blood and to remove unwanted cells. After flushing, 1 mL of ice-cold acetone is introduced at the flow rate of 2.5 mL/hr to the channel to fix cancer cells onto the substrate. The sample slide is then disassembled and dried and stored in fridge (4°C) before staining. Detailed introduction of the experimental and fluorescent staining protocols can be found in our previous reports^11^.

### Blood specimen collection

Blood samples are drawn from multiple healthy donors after obtaining informed consent under an IRB-approved protocol. Written informed consents were obtained from all participants. This study was approved by the Institutional Biosafety Committee (IBC) and the Advisory Committee on Human Research at the University of Texas at Austin. All experiments were performed in accordance with the declaration of Helsinki. All specimens are collected and stored in CellSave tubes (*Veridex, Janssen Diagnostic, J&J*).

### Capture rate

The capture rate is defined as follows: when preparing the cell suspension for the spiked sample, the same amount of cell suspension is dropped on two glass slides and used as control samples. The capture rate is calculated by dividing the number of cells found from the spiked samples by the average number of cells found on the control slides. Since the number of cells in each aliquot cannot be very accurately known, chances are that more cells are spiked into the blood samples than the control slides, which could result in a nominal over 100% capture rate. One can normalize the data to 100%, but we choose to present the original data for comparison.

### Statistical analysis

Data are reported as mean ± standard deviation of the mean as noted. Assuming groups have a normal distribution and homogenous variances, the group means are compared by an independent two sample T-test, and the variances are compared using a Levene's test. Differences are considered significant at the 95% confidence level (p < 0.05).

## Author Contributions

X.J.Z. and K.H. initiated the concept and design of microchip immunomagnetic assay of CTCs. P.C. designed the micromagnets and conducted the theoretical studies. Y.-Y.H. performed the experimental studies. P.C., Y.-Y.H., K.H. and X.J.Z. analyzed the results. All authors participated in the writing and revisions of the manuscript.

## References

[b1] Turner N. *et al.* Can biomarker assessment on circulating tumor cells help direct therapy in metastatic breast cancer? Cancers. 6, 684–707 (2014). 24670368 10.3390/cancers6020684 PMC4074798

[b2] Gupta G. P. & Massagué J. Cancer Metastasis: Building a Framework. Cell. 127, 679–695 (2006). 17110329 10.1016/j.cell.2006.11.001

[b3] Bidard F.-C. *et al.* Clinical application of circulating tumor cells in breast cancer: overview of the current interventional trials. Cancer. Metast. Rev. 32, 179–188 (2013). 10.1007/s10555-012-9398-0 PMC3655223 23129208

[b4] Yu M. *et al.* Circulating Breast Tumor Cells Exhibit Dynamic Changes in Epithelial and Mesenchymal Composition. Science. 339, 580–584 (2013). 23372014 10.1126/science.1228522 PMC3760262

[b5] Powell A. A. *et al.* Single Cell Profiling of Circulating Tumor Cells: Transcriptional Heterogeneity and Diversity from Breast Cancer Cell Lines. PLOS ONE. 7, e33788 (2012). 22586443 10.1371/journal.pone.0033788 PMC3346739

[b6] Nagrath S. *et al.* Isolation of rare circulating tumour cells in cancer patients by microchip technology. Nature. 450, 1235–1239 (2007). 18097410 10.1038/nature06385 PMC3090667

[b7] Hosokawa M. *et al.* Size-Selective Microcavity Array for Rapid and Efficient Detection of Circulating Tumor Cells. Anal. Chem. 82, 6629–6635 (2010). 20583799 10.1021/ac101222x

[b8] Shim S., Gascoyne P., Noshari J. & Stemke Hale K. Dynamic physical properties of dissociated tumor cells revealed by dielectrophoretic field-flow fractionation. Integr. Biol. 3, 850–862 (2011). 10.1039/c1ib00032b PMC3864024 21691666

[b9] Gossett D. *et al.* Label-free cell separation and sorting in microfluidic systems. Anal. Bioanal. Chem. 397, 3249–3267 (2010). 20419490 10.1007/s00216-010-3721-9 PMC2911537

[b10] Gossett D. R. *et al.* Inertial Manipulation and Transfer of Microparticles Across Laminar Fluid Streams. Small. 8, 2757–2764 (2012). 22761059 10.1002/smll.201200588

[b11] Hoshino K. *et al.* Microchip-based immunomagnetic detection of circulating tumor cells. Lab Chip. 11, 3449–3457 (2011). 21863182 10.1039/c1lc20270g PMC3379551

[b12] Huang Y.-Y. *et al.* Immunomagnetic nanoscreening of circulating tumor cells with a motion controlled microfluidic system. Biomed. Microdevices. 15, 673–681 (2013). 23109037 10.1007/s10544-012-9718-8 PMC3584207

[b13] Zborowski M., Fuh C. B., Green R., Sun L. & Chalmers J. J. Analytical magnetapheresis of ferritin-labeled lymphocytes. Anal. Chem. 67, 3702–3712 (1995). 8644920 10.1021/ac00116a014

[b14] Liang L., Zhang C. & Xuan X. Enhanced separation of magnetic and diamagnetic particles in a dilute ferrofluid. Appl. Phys. Lett. 102, 234101–234104 (2013).

[b15] Probst C. E., Zrazhevskiy P. & Gao X. Rapid Multitarget Immunomagnetic Separation through Programmable DNA Linker Displacement. J. Am. Chem. Soc. 133, 17126–17129 (2011). 21988124 10.1021/ja2072324 PMC3208664

[b16] Zhu T. *et al.* Continuous-flow ferrohydrodynamic sorting of particles and cells in microfluidic devices. Microfluid. Nanofluid. 13, 645–654 (2012). 10.1007/s10404-012-1004-9 PMC4587988 26430394

[b17] Chen P., Huang Y.-Y., Hoshino K. & Zhang X. Multiscale immunomagnetic enrichment of circulating tumor cells: from tubes to microchips. Lab Chip. 14, 446–458 (2014). 24292816 10.1039/c3lc51107c

[b18] Fanelli C. *et al.* Magnetic fields increase cell survival by inhibiting apoptosis via modulation of Ca2+ influx. FASEB J. 13, 95–102 (1999). 9872934 10.1096/fasebj.13.1.95

[b19] Kimura T., Yamato M. & Nara A. Particle trapping and undulation of a liquid surface using a microscopically modulated magnetic field. Langmuir. 20, 572–574 (2004). 15773077 10.1021/la035768m

[b20] Lee C., Lee H. & Westervelt R. Microelectromagnets for the control of magnetic nanoparticles. Appl. Phys. Lett. 79, 3308–3310 (2001).

[b21] Lee H., Purdon A. & Westervelt R. Manipulation of biological cells using a microelectromagnet matrix. Appl. Phys. Lett. 85, 1063–1065 (2004).

[b22] Hsieh D., Langer R. & Folkman J. Magnetic modulation of release of macromolecules from polymers. Proc. Natl. Acad. Sci. 78, 1863–1867 (1981). 6940193 10.1073/pnas.78.3.1863 PMC319235

[b23] Steiner U. E. & Ulrich T. Magnetic field effects in chemical kinetics and related phenomena. Chem. Rev. 89, 51–147 (1989).

[b24] Inglis D. W., Riehn R., Austin R. H. & Sturm J. C. Continuous microfluidic immunomagnetic cell separation. Appl. Phys. Lett. 85, 5093–5095 (2004).

[b25] Deng T., Prentiss M. & Whitesides G. M. Fabrication of magnetic microfiltration systems using soft lithography. Appl. Phys. Lett. 80, 461–463 (2002).

[b26] Nawarathna D. *et al.* Shrink-induced sorting using integrated nanoscale magnetic traps. Appl. Phys. Lett. 102, 063504-063504-063505 (2013). 10.1063/1.4790191 PMC3585756 23479497

[b27] Dumas-Bouchiat F. *et al.* Thermomagnetically patterned micromagnets. Appl. Phys. Lett. 96, 102511-102511-102513 (2010).

[b28] Zanini L. F., Dempsey N. M., Givord D., Reyne G. & Dumas-Bouchiat F. Autonomous micro-magnet based systems for highly efficient magnetic separation. Appl. Phys. Lett. 99, 232504-232504-232503 (2011).

[b29] Zheng S. *et al.* 3D microfilter device for viable circulating tumor cell (CTC) enrichment from blood. Biomed. Microdevices. 13, 203–213 (2011). 20978853 10.1007/s10544-010-9485-3 PMC3809998

[b30] Park S. *et al.* Morphological Differences between Circulating Tumor Cells from Prostate Cancer Patients and Cultured Prostate Cancer Cells. PLOS ONE. 9, e85264 (2014). 24416373 10.1371/journal.pone.0085264 PMC3885705

[b31] Wu C.-H. *et al.* Versatile immunomagnetic nanocarrier platform for capturing cancer cells. ACS nano 7, 8816–8823 (2013). 24016305 10.1021/nn403281e PMC3846426

[b32] Wang S. *et al.* Highly efficient capture of circulating tumor cells by using nanostructured silicon substrates with integrated chaotic micromixers. Angew. Chem. Int. Ed. 50, 3084–3088 (2011). 10.1002/anie.201005853 PMC3085082 21374764

[b33] Mitchell M. J., Wayne E., Rana K., Schaffer C. B. & King M. R. TRAIL-coated leukocytes that kill cancer cells in the circulation. Proc. Natl. Acad. Sci. 111, 930–935 (2014). 24395803 10.1073/pnas.1316312111 PMC3903223

[b34] Hoshino K., Chen P., Huang Y.-Y. & Zhang X. Computational Analysis of Microfluidic Immunomagnetic Rare Cell Separation from a Particulate Blood Flow. Anal. Chem. 84, 4292–4299 (2012). 22510236 10.1021/ac2032386 PMC3359653

[b35] Judy J. W. & Muller R. S. Magnetic microactuation of torsional polysilicon structures. Sens. Actuators, A. 53, 392–397 (1996).

[b36] Crangle J. & Goodman G. M. The Magnetization of Pure Iron and Nickel. Proc. R. Soc. London, Ser. A. 321, 477–491 (1971).

[b37] Suresh S. Biomechanics and biophysics of cancer cells. Acta Mater. 55, 3989–4014 (2007). 10.1016/j.actbio.2007.04.002 PMC2917191 17540628

[b38] Stott S. L. *et al.* Isolation of circulating tumor cells using a microvortex-generating herringbone-chip. Proc. Natl. Acad. Sci. 107, 18392–18397 (2010). 20930119 10.1073/pnas.1012539107 PMC2972993

[b39] Stroock A. D. *et al.* Chaotic mixer for microchannels. Science. 295, 647–651 (2002). 11809963 10.1126/science.1066238

[b40] Furlani E. P. & Sahoo Y. Analytical model for the magnetic field and force in a magnetophoretic microsystem. J. Phys. D: Appl. Phys. 39, 1724 (2006).

[b41] Huang Y.-Y. *et al.* Patterned nanomagnets on-chip for screening circulating tumor cells in blood. Proceedings of MicroTAS. 2, 1117–1119 (2012).

